# Hybridization of Two Major Termite Invaders as a Consequence of Human Activity

**DOI:** 10.1371/journal.pone.0120745

**Published:** 2015-03-25

**Authors:** Thomas Chouvenc, Ericka E. Helmick, Nan-Yao Su

**Affiliations:** 1 Department of Entomology and Nematology, Fort Lauderdale Research and Education Center, University of Florida, Institute of Food and Agricultural Sciences, Fort Lauderdale, Florida, United States of America; 2 Department of Plant Pathology, Fort Lauderdale Research and Education Center, University of Florida, Institute of Food and Agricultural Sciences, Fort Lauderdale, Florida, United States of America; Universidad Nacional Autonoma de Mexico, MEXICO

## Abstract

While hybridization of an invasive species with a native species is a common occurrence, hybridization between two invasive species is rare. Formosan subterranean termites (*Coptotermes formosanus*) and Asian subterranean termites (*C. gestroi*) are both ecologically successful and are the two most economically important termite pests in the world. Both species have spread throughout many areas of the world due to human activity; however, their distributions overlap in only three narrow areas because of distinct ecological requirements. In south Florida, where *C. formosanus* and *C. gestroi* are both invasive, the dispersal flight seasons of both species overlapped for the first time on record in 2013 and 2014. Pairings of heterospecific individuals were readily observed in the field and *C. gestroi* males preferentially engaged in mating behavior with *C. formosanus* females rather than females from their own species. In the laboratory, heterospecific and conspecific pairings had an equal colony establishment rate, but heterospecific incipient colonies had twice the growth rate of conspecific incipient colonies, suggesting a potential case of hybrid vigor. As all pre-zygotic barriers were lifted between the two species in the field, the apparent absence of post-zygotic barriers in the laboratory raises the possibility for introgressive hybridization in south Florida. While laboratory observations remain to be confirmed in the field, and the alate hybrid fertility is currently unknown, our results raise a tangible concern about the hybridization of two major destructive pest species. Such hybridization would likely be associated with a new economic impact.

## Introduction

Habitat alteration and human transportation have favored the spread of species with invasive capabilities as they may easily adapt to modified niches [1–3]. The establishment of invasive organisms in non-native areas can result in heterospecific interactions between invasive and native species, with potential for hybridization [4]. Introgression resulting from such hybridization can have important ecological and evolutionary consequences on native populations [5–8], and often are facilitated by human activity [4,9]. There is also mounting evidence that warming environments resulting from climate change can be an important factor contributing to such hybridization, either by altering the species distribution, or temporally shifting the mating season of species [10].

While the hybridization of non-native species with native species has been documented in a wide range of organisms [11,12], including plants [13], amphibians [14], fishes [15], mammals [16] and insects [17], few cases of hybridization involving two invasive species in non-native areas have been described. One such case is the hybridization of two invasive fire ant species (*Solenopsis invicta × S. richteri*) where a hybrid zone is now fully established in the Southern United States [18,19]. To a lesser extent, gene introgression from the Africanized honey bee to European honey bee populations (*Apis mellifera* subspecies) has become a problem for human activity in North and South America [20]. Hybrid introgressions among non-native organisms have only been described in a couple of social insects with socioeconomic impacts. Here, we describe a potentially new case of hybridization between two invaders in another social insect group with major economic importance.

Many subterranean termite (Isoptera: Rhinotermitidae) species are considered “urban pests” due to their tendency to attack man-made structures [21], and some are now invasive throughout the world, increasingly causing structural damage [22]. The Formosan subterranean termite (*Coptotermes formosanus*) and the Asian subterranean termite (*C. gestroi*) are the two most destructive structural pests in the world and are responsible for most of the $40 billion annual economic impact from termite damage [21]. As social insects, mature *Coptotermes* colonies can reach more than a million individuals [23] with caste polymorphism and polyethism [24], and have underground foraging galleries reaching up to 100 m, making detection and control difficult [25,26].

**Fig 1:**
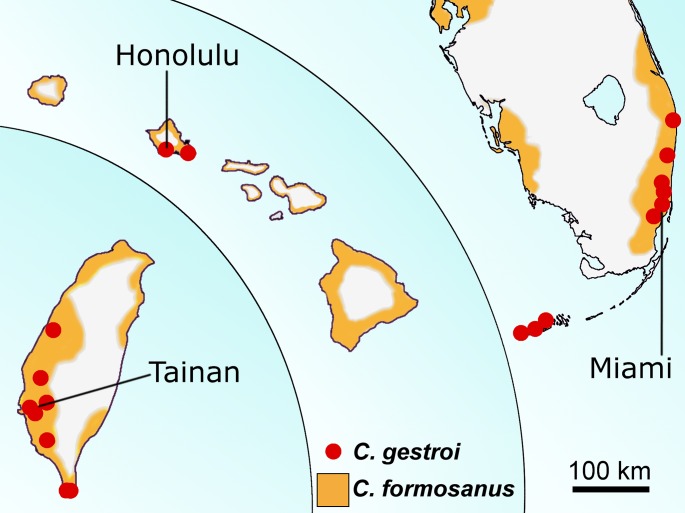
The distributions of *C. formosanus* and *C. gestroi* overlap in three areas in the word. From left to right: Taiwan, Hawaii, south Florida, according to [32,33] (modified for illustrative purpose only).

*Coptotermes formosanus* is endemic to China and Taiwan and has spread to many temperate and subtropical regions of the world [22]. It is now found throughout the southeastern United States and is responsible for more than $1 billion of structural damage each year in the United States alone [27]. *Coptotermes gestroi* is native to southeast Asia and has spread in many tropical regions, being potentially the most ubiquitous and destructive subterranean termite pest in the world [22]. Both species have distinct ecological requirements [28], but there are now established populations in many non-native areas due to human activity [29]. This observation reflects the current global biotic homogenization of some ecosystems, i.e. the replacement of native biotas by a small group of expanding non-native species in many parts of the world [30,31]. Their distributions now overlap in three narrow locations of the world [32,33]: the south part of the island of Taiwan, the island of Oahu in Hawaii, and southeast Florida (Fig. 1). However, studies concerning the interaction between *C. formosanus* and *C. gestroi* are restricted to competition between workers and soldiers from mature colonies, where individuals displayed interspecies agonism and competed for the access to resources [28,34]. The interspecies interactions of individuals from the reproductive caste (alates) have not yet been investigated.

Swarming events (dispersal flights) in *Coptotermes* consist of mature colonies seasonally emitting thousands of alates at dusk [35]. Individuals drop their wings after the dispersal flight, find a mate, and engage in tandem behavior. The tandem behavior is initiated by the male as he maintains contact with the tip of the female’s abdomen [36]. The female then leads the way in search of a favorable nesting site, in which both individuals seclude themselves and establish the incipient colony [37]. It takes up to 8 yrs for these colonies to mature and initiate dispersal flights [38]. In south Florida, it was documented that the two species have distinct dispersal flight seasons [35]; however, for the first time on record, our monitoring of both species showed that alates swarmed simultaneously in a single location (Ft. Lauderdale, Florida, USA) on March 20^th^ and April 3^rd^ 2013.

Our preliminary observation of simultaneous swarming in 2013 implies that both geographical and temporal isolations may now be lifted between alates of *C. formosanus* and *C. gestroi*, giving the opportunity for interspecies mating. Because both species presumably went through allopatric divergence (different native distribution and ecological requirements), we hypothesized that the absence of reinforcement [39] may have prevented the formation of behavioral or physiological barriers against hybridization. We therefore investigated if pre-zygotic and post-zygotic barriers between *C. formosanus* and *C. gestroi* were lifted, and discussed the possibilities for the establishment of a hybrid termite population in areas where both species are established.

## Results

### (a) Swarming seasons overlap and tandem behavior

**Fig 2:**
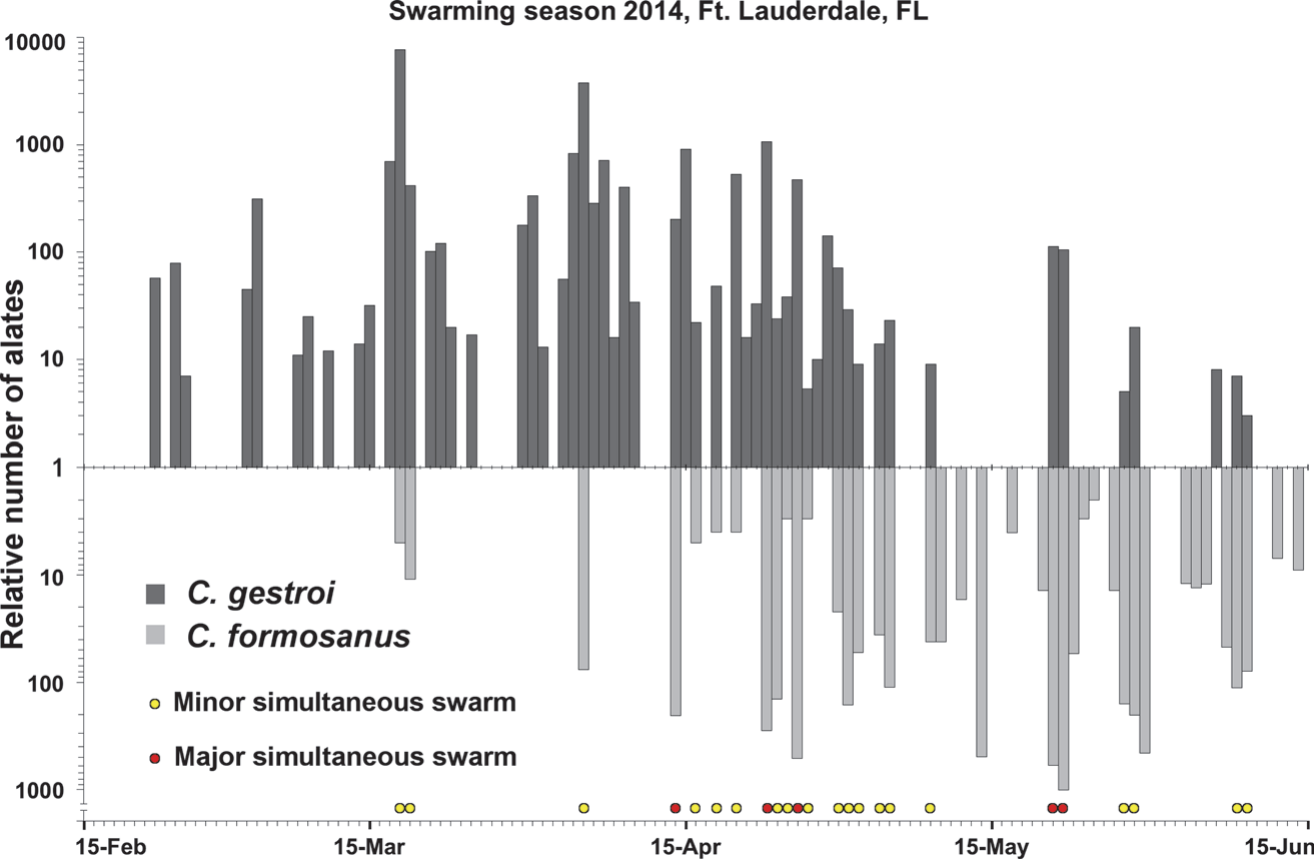
In south-east Florida, the dispersal flights (swarming seasons) of both species overlapped in 2014. “Major simultaneous swarming event” indicates that more than 100 individuals were collected this day while “minor simultaneous swarming event” indicates that fewer than 100 individuals were collected for at least one of the species. Between March 18^th^ and June 9^th^, there were 24 events of simultaneous *C. formosanus* and *C. gestroi* dispersal flights including 5 major simultaneous swarming events. The relative number of alates collected every day is presented on a log scale.

In 2013, simultaneous swarming events of *C. formosanus* and *C. gestroi* alates were casually observed on two distinct days (March 20^th^ and April 3^rd^) at dusk in Ft. Lauderdale (Florida, USA). On those combined two days, 20–40 individuals from each sex were collected for both species and used for establishing incipient colonies in the laboratory. In 2014, the daily monitoring of swarming events at dusk, between February 15^th^ and June 15^th^ (120 d), resulted in observation of 55 swarming events for *C. gestroi* alates and 40 swarming events for *C. formosanus* alates at the same location. During this period, 20,018 *C. gestroi* alates and 5,001 *C. formosanus* alates were collected. There were 24 events of simultaneous dispersal flights between March 18^th^ and June 9^th^, including five major simultaneous swarming events (Fig. 2), which confirmed the overlap of swarming seasons previously observed in 2013.

**Fig 3:**
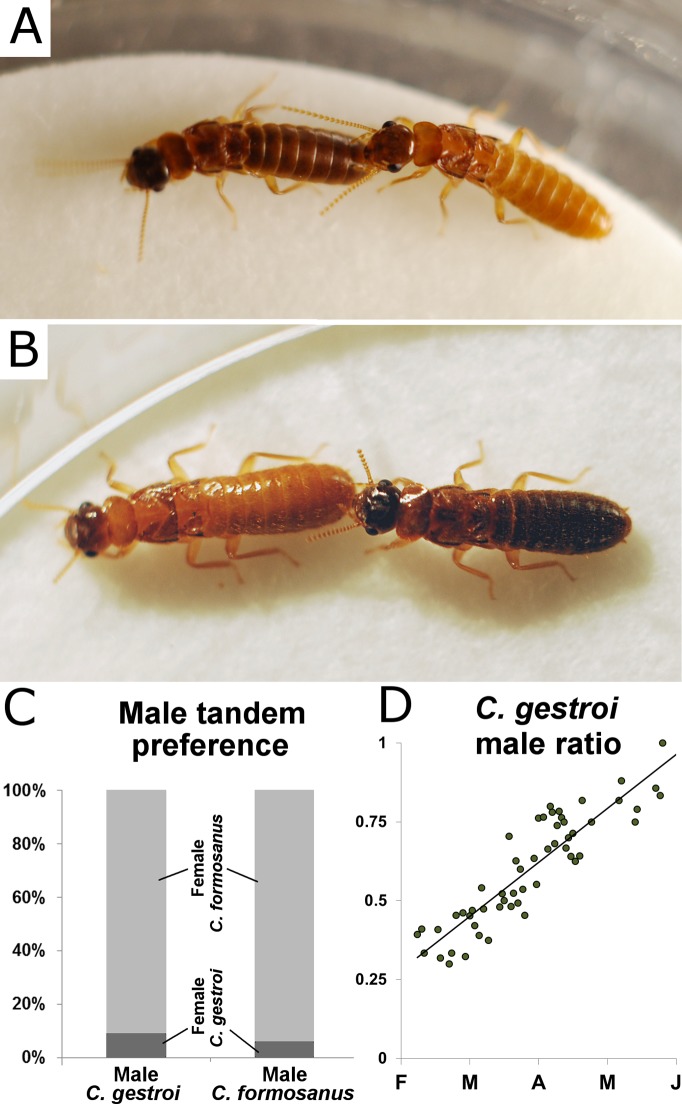
Interspecies tandem. (A) male *C. formosanus* maintaining tandem with female *C. gestroi* (B) male *C. gestroi* maintaining tandem with female *C. formosanus* (C) Tandem preference in a choice test, where male *C. gestroi* and male *C. formosanus* primarily initiated tandem behavior with female *C. formosanus* (binomial test, 91% and 94% respectively, *p*<0.001 for both). (D) Sex ratio was male-biased (linear model, *F*_*(42)*_ = 133, *p*<0.001) for *C. gestroi* at the time of the swarming overlap, increasing the chance for male *C. gestroi* and female *C. formosanus* tandem formation in the field.

Males and females of both species readily initiated interspecies tandem behavior (Fig. 3A, 3B), with direct observations of interspecies tandems in the field (S1 Fig.). From a visual observation of termite tandems in the field on May 21^st^ 2014, we estimated that interspecies tandems represented approximately one quarter of all tandem observations (38 individual interspecies tandems on a 3 m × 3 m surface within 30min of observation). However, we only observed male *C. gestroi* initiating and maintaining tandem with female *C. formosanus* in the field. In a laboratory choice assay between a female of each species, male *C. gestroi* and male *C. formosanus* both preferentially initiated a tandem with female *C. formosanus* (binomial test, 91% and 94% respectively, *p*<0.001 for both, Fig. 3C). In addition, toward the end of the *C. gestroi* swarming season, which corresponded to the peak of overlap of dispersal flights (April 15^th^—May 20^th^), *C. gestroi* sex-ratio was strongly male biased (Fig. 3D, linear model, *F*_*(42)*_ = 133, *p*<0.001), increasing the chance for female *C. formosanus* and male *C. gestroi* interactions in the field during simultaneous swarms.

### (b) Development of incipient hybrid colonies

**Fig 4:**
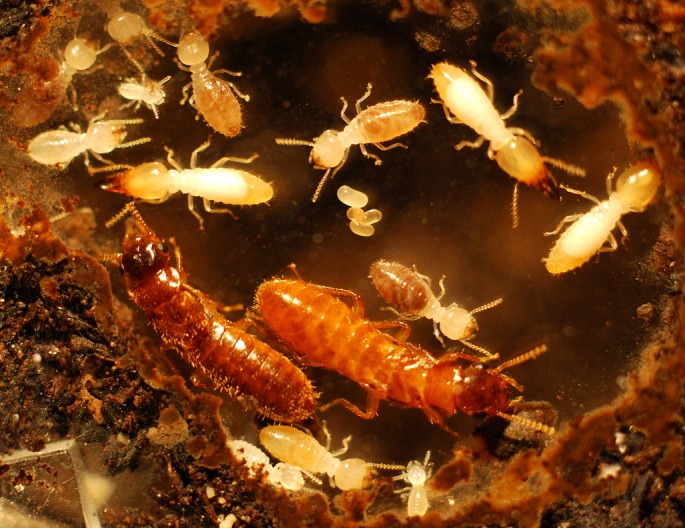
Heterospecific colonies have viable hybrid offspring. Shown here is a eight month-old incipient colony that contains the male *C. gestroi*, the female *C. formosanus*, eggs, larvae, workers and soldiers.

Incipient colonies were established in 2013 in the laboratory by pairing a male and a female with four possible combinations, conspecific colonies (20 ♀*C. gestroi* × ♂*C. gestroi*, 18 ♀*C. formosanus* × ♂*C. formosanus*) and heterospecific colonies (10 ♀*C. gestroi* × ♂*C. formosanus*, 18 ♀*C. formosanus* × ♂*C. gestroi*) in individual rearing units. The initial colony development in all units was similar to what was previously described in *C. formosanus* incipient colonies [24]: the first eggs were observed within 25 d in most units and the first instar larvae developed within 40 d. First instar workers were observed within 65 d, and the first soldier was observed within 75 d (Fig. 4). Soldiers from mature colonies of *C. gestroi* and *C. formosanus* can be morphologically distinguished [40], however in incipient termite colonies, soldiers are produced from an accelerated developmental pathway (i.e. nanitic soldiers) and none of the soldiers displayed traits characteristic of either species [41].

**Fig 5:**
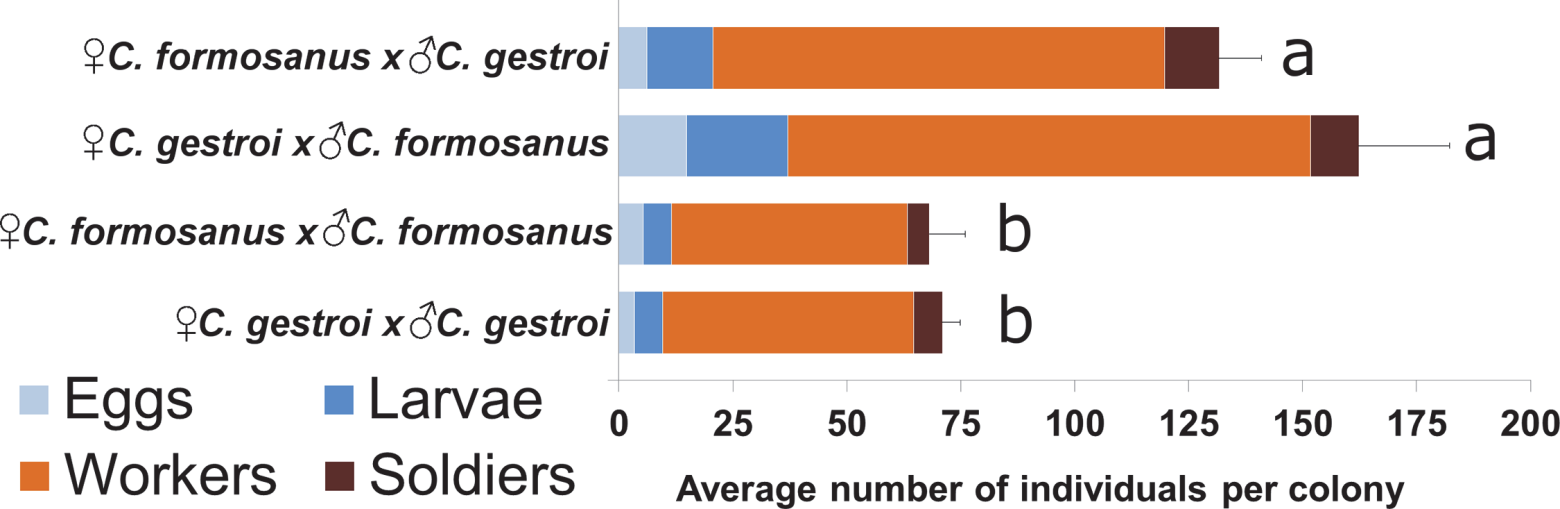
After one year of development, heterospecific colonies have average 2.03 times the number of individuals than conspecific colonies (*F*_*(3*,*32)*_ = 24.21, *p*<0.001, HSD post-hoc, same letter in a category indicates no significant difference).

After one year of colony development, there was no significant difference in the failure of incipient colonies to establish between conspecific and heterospecific colonies (average colony mortality = 50%, typical under laboratory conditions [37], *χ* ^*2*^ = 0.711, *dl* = 3, *p* = 0.87). However, one-year old heterospecific colonies displayed faster development (×2.03 the number of individuals produced in average) than conspecific colonies (*F*_*(3*,*32)*_ = 24.21, *p*<0.001, Fig. 5). There was no difference in the proportion of castes and developmental stages among the four mating combinations after one year of development (*χ* ^*2*^ test of independence, *χ* ^*2*^ = 4.64, *dl* = 9, *p* = 0.86), with an average proportions of 6.8% eggs, 11.4% larvae, 74.1% workers and 7.7% soldiers.

### (c) Molecular confirmation of the hybridization

Eight colonies (two per mating combination) were processed for genetic analysis. Each parental species displayed a single specific COII genotype, as previously described by [32]. For heterospecific colonies, the offspring inherited maternal mitochondrial markers, with ♀*C. gestroi* × ♂*C. formosanus* offspring displaying the *C. gestroi* COII genotype, and with ♀*C. formosanus* × ♂*C. gestroi* offspring displaying the *C. formosanus* COII genotype. Similarly, offspring from conspecific colonies displayed the COII type of their respective species (S1 Table).

**Fig 6:**
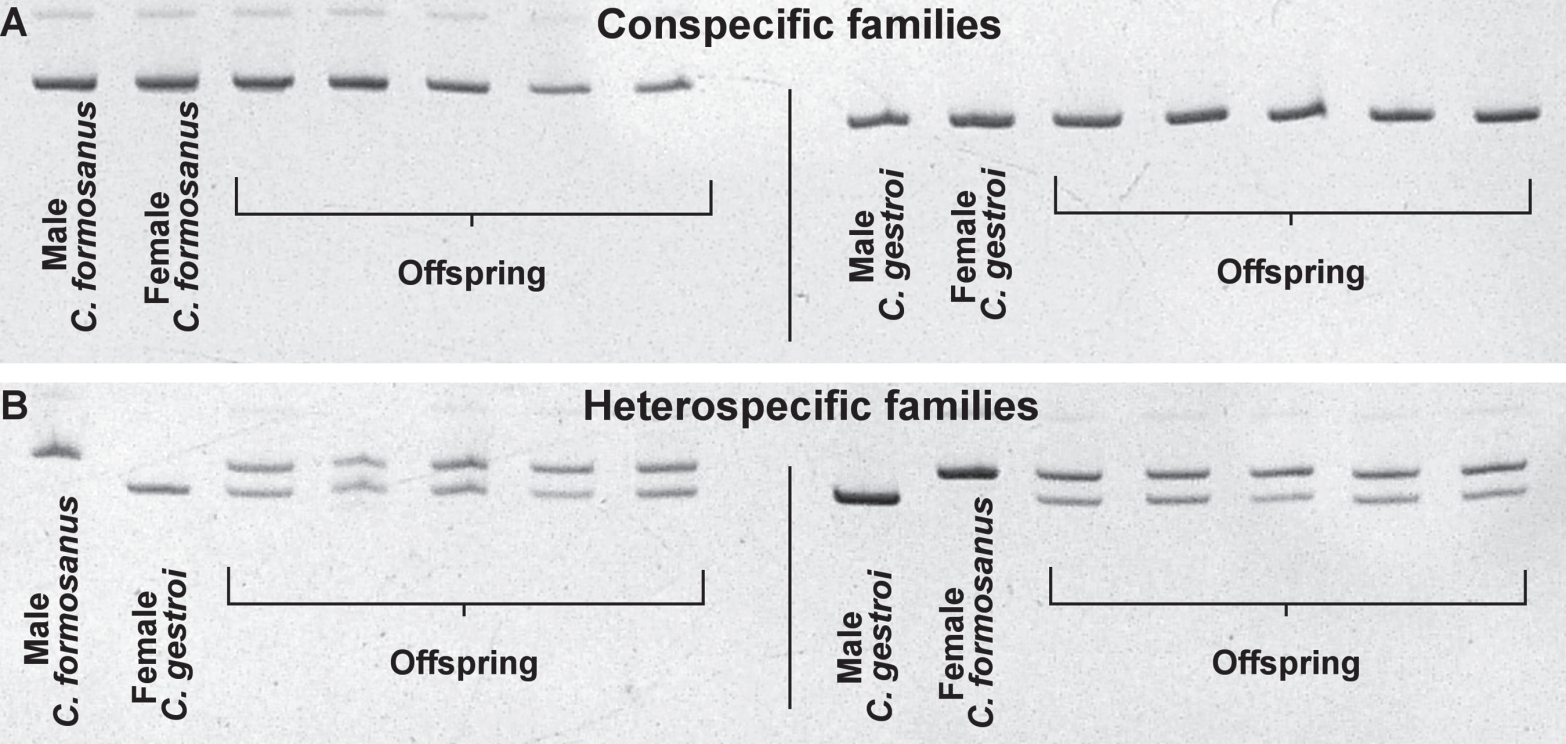
Results of the microsatellite *Cg33* locus for the male, the female and five random offspring from the four mating combinations, where (A) conspecific colonies are homozygous for a species specific allele, and (B) heterospecific colonies have offspring displaying alleles from both species, confirming the hybridization.

Microsatellite analysis for the *Cg33* and *Clac1* loci showed that all individuals from conspecific colonies were homozygous for both loci with species specific alleles (S2 Fig., S3 Fig.). We finally confirmed the hybridization of the offspring from heterospecific colonies (Fig. 6) as all individuals were heterozygous for *Cg33* and *Clac1* with alleles from both *C. gestroi* and *C. formosanus* origin (S2 Fig., S3 Fig.).

## Discussion

Our laboratory results indicate that *C. formosanus* and *C. gestroi* populations established in south Florida have the potential for the hybridization between two invasive species. The overlapping distribution and dispersal flight seasons in 2013 and 2014 provided the opportunity for male and female alates of the two species to interact directly. Interspecies mating was observed in the field and confirmed in the laboratory. All observations therefore indicate that there is currently no pre-zygotic barrier that may prevent hybridization between *C. formosanus* and *C. gestroi* in south Florida (S4 Fig.). The survival rate of hybrid colonies in the laboratory was not significantly different than that of conspecific colonies of the parental species. Although it was not possible to monitor the survival of incipient colonies in the field, our laboratory results suggest that heterospecific colonies have the potential for successfully establishing in the field. The genetic analysis of incipient colonies confirmed that individuals produced by heterospecific pairs were the result of hybridization, which excluded a potential case of parthenogenesis [42] or the unlikely storage of sperm by females from a previous mating [43]. Finally, hybrid colonies in the laboratory displayed high vigor by producing twice the number of individuals compared to incipient conspecific colonies.

At this point, it is premature to determine if any post-zygotic barriers would further prevent the hybridization process, but there are possible scenarios emerging from established heterospecific colonies. 1) Despite high colony vigor at the incipient stage, heterospecific colonies may later display maladaptive genotypes that would prevent them from reaching maturity or produce alates, eliminating all hybridization possibilities. 2) If produced, alates from heterospecific colony may be sterile, genetically or phenotypically incompatible with parental species, preventing any gene introgression back into the parental species. 3) If viable and fertile, alate hybrids may have limited overlapping swarming seasons with parental species, hindering the gene introgression process. 4) Finally, in the case of absence of post-zygotic barriers, with fully functional alate hybrids and their offspring, hybridization and introgression would be unavoidable.

Because male *C. gestroi* preferentially paired with female *C. formosanus*, we suggest that males of both species responded to the same cue for female attractiveness (presumably an identical sexual pheromone) but that female *C. formosanus* is a super releaser in comparison to female *C. gestroi*. In addition, the strong male-bias swarms of *C. gestroi* during the overlapping season also increased the chance for female *C. formosanus* and male *C. gestroi* mating combinations to found new colonies in the field. Such mating bias implies that in the scenario where all pre-zygotic and post-zygotic barriers were lifted, hybridization in the field would be unidirectional, resulting in an asymmetrical introgression of *C. gestroi* genes into *C. formosanus* populations.

There have been few studies regarding hybridization in termites and these have mostly described gene flow among populations of subspecies in the lower termites, *Reticulitermes lucifugus* [44] and *Zootermopsis nevadensis* [45], with cases of introgressive hybrids. In higher termites, no hybrid viability was observed between two species of *Pseudacanthotermes* [46], but some hybrid viability was observed in laboratory between two species of *Nasutitermes* for 60 d [47]. All cases with viable F_1_ occurred among termite populations with little genetic divergence (<30 substitutions in the COII gene). In comparison, Floridian populations of *C. gestroi* and *C. formosanus* have 78 substitutions for the COII gene [32] implying further genetic distant between both species than any previously observed hybridization in termites.

One of the paradoxes of invasive species is that they can be highly successful despite low levels of genetic diversity and social insects are good representatives of such a phenomenon [48]. Because both *C. gestroi* and *C. formosanus* were introduced in south Florida within the past 30 years [49], possibly with single introduction events, there were cycles of inbreeding that resulted in a relative absence of genetic diversity in such populations [50,51]. The two south Florida *Coptotermes* populations were therefore isolated from gene flow of their respective native genetic pool, and the accumulation of homozygous alleles may have resulted in sub-optimal vigor (inbreeding depression) [52]. It was documented that *C. formosanus* incipient colonies from their native range in China could reach more than 200 individuals within the first year [53], while in average we observed 70 individuals in our *C. formosanus* incipient colonies. The high numbers of individuals observed in hybrid colonies may reflect a case of heterosis (restoration of heterozygosity) [54], but such observation requires to be confirmed in the following years.

With a subtropical climate and strong human activity, the continuous spread of exotic termites in south Florida is inevitable [35]. Climate change can directly shift the distribution range [55] and the timing of reproduction of species [56] because of alterations of environmental conditions [57], and hybridization in animals has been observed as a consequence of shifts in species distributions due to climate change [10,58]. The unusually warm 2013 and 2014 winters (5^th^ and 10^th^ warmest winter on record) with successive cold fronts in the region (Source: NOAA) may have allowed for a wide overlap of the termite species dispersal flight seasons. In the scenario that such weather patterns become frequent in the near future [59], with the *C. gestroi* range predicted to move further north [60], simultaneous swarms may become a common occurrence. The documented case of introgressive hybridization in fire ants (*Solenopsis*) in the Southern United States [61] may serve as a cautionary tale, and the establishment of a hybridized *Coptotermes* population in south Florida with continuous gene flow between the two species remains a possibility. Both species have been established in Hawaii and Taiwan for a longer time [28,62], but hybridization has not been documented yet. The opportunity for *C. formosanus* and *C. gestroi* to hybridize may be unique to south Florida due to particular environmental cues and overlapping swarming seasons, but monitoring the dispersal flights in Hawaii and Taiwan would provide insight into the possibility of interspecies mating in all three locations.

To conclude, this study showed that all pre-zygotic barriers were lifted between *C. formosanus* and *C. gestroi* in the field, and high hybrid vigor was obtained in the laboratory. In the hypothetical scenario that no further post-zygotic barriers would prevent the hybridization, faster colony development in interspecific colonies (as observed in laboratory) would allow such colonies to reach maturity earlier and possibly disperse faster than the parental *C. formosanus* and *C. gestroi* colonies. Currently, it is not known if a hybrid colony can produce fertile and fully functional alates for maintaining hybrid populations and for possible introgressive hybridization back into the *C. formosanus* and *C. gestroi* populations (S4 Fig.). Because of the long life cycle of *Coptotermes*, it may take from 5 to 8 years before colonies mature in the laboratory and in the field [24,38], which would enable us to confirm the fertility of the F_1_ generation. In addition, due to the cryptic habit of termites and the currently unknown morphology of hybrid alates and soldiers from mature heterospecific colonies, it may take several years before the hybridization could be effectively monitored and confirmed in the field. *Coptotermes* mature colonies can contain millions of individuals and live up to 20 yrs [24] and even in the absence of alate hybrid fertility, the persistence of hybrid colonies in urban environments would still present a threat to structures (i.e., a kick from a mule is as good as a kick from a donkey).

## Materials and Methods

### (a) Termite collection and ethic statement

No specific permits were required for the termite’s collection from the field and for maintenance in laboratory. No endangered or protected species were involved in this study. All termites used for incipient colonies in this study for *C. formosanus* and *C. gestroi* were obtained from swarming events collected in Broward County (Florida, USA) in a privately owned, non-protected location.

In spring 2013, *C. formosanus* and *C. gestroi* alates were casually collected in Ft. Lauderdale, FL (26.105°N, 80.175°W) at dusk between March 15^th^ and April 4^th^ using a fluorescent light and an insect net. We obtained the first record of simultaneous *C. formosanus* and *C. gestroi* swarms during this time, as it was previously established that both species had distinct dispersal flight seasons [35]. However, we could only partially describe the flight phenology of the two species in the area for the 2013 swarm season. To fully characterize the flight activity of both species throughout the season, a light trap was setup daily between Feb 15^th^ and June 15^th^ 2014 at the same location. Monitoring was initiated 30 minutes before sunset and terminated 90 min after sunset. The light trap was modified from Peppuy [63] using a 1,720 lumen LED work light in addition to a fluorescent dark light and a regular fluorescent light. A 3m × 3m plastic sheet was laid under the light trap to ease observations around the trap. As termites were attracted to the light trap, we were able to observe tandem behavior from some individuals that landed outside the trap (S1 Fig.). Inside the trap, small rolls of moist corrugated cardboard were placed for temporary nesting sites, into which termites freely moved and isolated themselves in single cells. Cardboard rolls were transferred to the laboratory the next morning and processed to count the collected individuals, determine their sex and their species, and establish incipient colonies. In this study, we defined a “major simultaneous swarming event” as when both species swarmed on a given day with more than 100 individuals of both species collected in the light trap. A “Minor simultaneous swarming event” implies that less than 100 individuals were collected for at least one of the species.

### (b) Tandem behavior assay

Tandem behavior is the process in which a male dealate follows a female dealate; the male antennae and mouthparts remain in contact with the female abdomen while she searches for a suitable nesting site [36,37]. Interspecies and intraspecies tandem behaviors were directly observed in the field on most evenings of simultaneous flights. In the laboratory, males were submitted to a choice test between a female of each species. One female *C. gestroi* and one female *C. formosanus* were placed in a 5 cm Petri dish with a moist filter paper on the bottom. One male *C. gestroi* or one male *C. formosanus* was introduced in the Petri dish and tandem behavior was monitored for 5 min (n = 100 males per species). If the male maintained a tandem position with a single female for at least 4 min (cumulative time), the female was marked as “preferred female.” (Note: as the males engaged in tandem behavior with a female of their choice, they maintained the tandem in 96% of the replicates. The eight remaining replicates were repeated to obtain a clear choice for all assays).

### (c) Incipient colonies and rearing conditions

Dealates of both species were paired to establish incipient colonies in the laboratory. One male and one female were introduced into rearing units [24,64]. However, because of the relatively small number of individuals collected in 2013, a limited number of colonies were established per mating combination: 20 ♀*C. gestroi* × ♂*C. gestroi*, 18 ♀*C. formosanus* × ♂*C. formosanus*, 10 ♀*C. gestroi* × ♂*C. formosanus*, and 18 ♀*C. formosanus* × ♂*C. gestroi*. In 2014, a large number of alates were collected, and we established 200 incipient colonies per mating combination and will be used for future studies.

A rearing unit was composed of a transparent plastic cylindrical vial (8 cm × 2.5 cm diameter, internal volume = 37 cm^3^) with 6 g of moistened organic soil at the bottom. Four blocks of *Picea sp*. (5 cm × 0.5 cm × 0.5 cm) were positioned vertically and an additional *Picea* block (10 cm × 0.5 cm × 0.5 cm) was placed inside the vial, along the vertical side. A 3% agar solution was poured, leaving a 2-cm space at the top of the vial. When the agar was solidified, the long *Picea* block was removed from the vial to leave a hole in the agar, providing direct access to the soil on the bottom and to the wood. A perforated plastic cap was placed on the top to allow for aeration, but to limit desiccation and prevent escape. Rearing units were stored at 28°C for 365 d. A small amount of water was periodically added to vials that showed signs of dryness. After one year, all rearing units were opened to count for individuals of each caste and developmental stage (primary reproductives, eggs, larvae, workers and soldiers) and colonies that possessed a live male and female with their offspring were considered “successful colonies”.

### (d) Sample preparation for DNA extractions

Two incipient colonies of each pairing combination (see “*Incipient colonies and rearing conditions*” above) were sacrificed for DNA extraction and subsequent polymerase chain reaction (PCR) for mitochondrial COII gene and microsatellites amplification. DNA was extracted from the parental male and female, 9 individual workers and one soldier from each of the incipient colonies using the Wizard DNA Extraction Kit (Promega, Inc.) per the manufacturer’s protocol, with the exception of the addition of GlycoBlue Coprecipitant (Invitrogen) for visualization of the DNA pellet. All DNA extraction concentrations were determined using a Qubit Fluorometer (Invitrogen) per the manufacturer’s protocol. DNA was stored at 4°C until ready for use.

### (e) Mitochondrial COII PCR conditions

Mitochondrial COII gene amplification for 12 of the individuals extracted from each of the incipient colonies was completed using previously published mitochondrial COII primers [32]. Forward primer A-tLeu (5’-ATGGCAGATTAGTGCAATGG-3’) and reverse primer B-tLys (5’-GTTTAAGAGACCAGTACTTG-3’) were used to amplify *C. formosanus*; and forward primer C2F2 (5’-ATACCTCGACGWTATTCAGA-3) and reverse primer B-tLys were used to amplify *C. gestroi*, per reference [32] who indicated that using the A-tLeu/B-tLys primer combination for *C. gestroi* resulted in the amplification of a COII pseudogene.

Total COII PCR reactions, 50 μL, contained final reagent concentrations of 10X PCR buffer [65], 1.5 mM MgCl_2_, 200 mM each dNTP, 0.4μM each primer, 1.25 U *Taq* DNA polymerase (New England Biolabs, Inc., Ipswich, MA), and sterile ultrapure water, 100 ng of extracted DNA was used as template for amplification. PCR thermocycling was carried out using either Mastercycler Gradient Thermocycler (Eppendorf North America, Hauppauge, NY) or Arktik Thermocycler (Thermo Fisher Scientific, Inc., Waltham, MA) under the following conditions: initial denaturation at 95°C for 90 sec followed by 34 cycles of denaturation at 95°C for 60 seconds, annealing at 55°C for 60 sec, extension at 72°C for 2 min; and final extension at 72°C for 10 min. 10 μL of the resulting PCR products were electrophoresed on a 1% agarose gel, stained with ethidium bromide (EtBr) and visualized using UV illumination. The remaining 40 μL of PCR products were cleaned using a Wizard DNA Clean-Up System (Promega, Madison, WI, USA), quantified using Qubit Fluorometer (Invitrogen) and sent to the University of Florida’s Core Genomics Facilities (ICBR, Gainesville, FL) for sequencing.

Mitochondrial COII forward/reverse sequences were assembled and edited using DNA Baser v2.9 [66]. COII primers for *C. gestroi* amplified a fragment of 932 bp while primers for *C. formosanus* amplified a fragment of 765 bp. Consensus sequences were aligned using MEGA version 6 [67] and identified by BLAST analysis through NCBI website (http://blast.st-va.ncbi.nlm.nih.gov/Blast.cgi); resulting sequences for *C. formosanus* and *C. gestroi* matched accession numbers EU805757 and EU805770, respectively, which are both Florida accessions deposited in GenBank and described by [32]. Offspring for all combinations of incipient colonies carried their respective maternal COII sequences (S1 Table).

### (f) Microsatellite genotyping

Twelve microsatellite markers previously described in various *Coptotermes* species [68–70] were investigated to determine whether any would be informative for cross-species amplification by testing them against a subset of individuals from the incipient colonies of *C. formosanus* and *C. gestroi* (S2 Table). Of the 12 microsatellite markers, *Cg33* [68] and *Clac1* [70] were the only two loci that successfully yielded product where each colony displayed homozygous species specific alleles. These microsatellites were used for further analyses of all incipient colony mating combinations.

Total microsatellite PCR reactions, 50 μL, contained final reagent concentrations of 10X PCR buffer [64], 1.5 mM MgCl 2, 200 mM each dNTP, 0.4μM each primer, 1.25 U *Taq* DNA polymerase (New England Biolabs, Inc., Ipswich, MA), and sterile ultrapure water, 100 ng of DNA extractions were used as template for amplification. PCR thermocycling was carried out using either Mastercycler Gradient Thermocycler (Eppendorf North America, Hauppauge, NY) or Arktik Thermocycler (Thermo Fisher Scientific, Inc., Waltham, MA) under the following conditions for primers *Cg33F/R* and *Clac1F/R*: initial denaturation at 95°C for 90 sec followed by 34 cycles of denaturation at 95°C for 30 seconds, annealing at 56°C/53°C for 60 sec, extension at 72°C for 2 min; and final extension at 72°C for 8 min. Five μL of the resulting PCR products were electrophoresed on an 8% polyacrylamide gel (PAGE), stained with ethidium bromide (EtBr) and visualized using UV illumination. The individuals with strong amplification were then used in a follow-up PCR reaction under the same parameters as above for each of the primers but using fluorescent 6FAM-tagged forward primers. Again, PCR’s were checked for amplification using 5 μL of the resulting PCR products as described above. The remaining 45 μL of PCR products were either diluted 1:10 or 1:100 with ultrapure water and a 20 μL aliquot was sent to the University of Florida’s Core Genomics Facilities (ICBR, Gainesville, FL) for genotyping.

Geneious 7.1.5 (Biomatters Ltd.) was used to assemble microsatellite files and to score and bin alleles for each of the incipient colonies. The microsatellites were scored using the 3^rd^ order least squares sizing method as recommended in the Geneious Microsatellite Plugin Manual (Biomatters Ltd.). Allele peaks with a Y-scale value of ≥8,000 were used in scoring alleles for all colonies; with the exception of some individuals where the initial PCR amplification itself was weak, these individuals alleles were scored if the Y-scale value was ≥4,000.

See S2 Fig. and S3 Fig. for microsatellite results. In brief, amplified locus *Cg33* yielded two alleles at 192 bp for *C. formosanus* and 210 bp for *C. gestroi*; and *Clac1* yielded 3 alleles at 173 or 183 bp in size for *C. formosanus* and 185 bp in size for *C. gestroi*. All offspring from the incipient colony pairings inherited one allele from each of the parental termites, confirming the hybrid status of the offspring.

### (g) Statistical analysis

The tandem choice test between *C. formosanus* and *C. gestroi* females was analyzed with a binomial test for each male species (n = 100 per species). The variation of the male sex ratio in *C. gestroi* dispersal fights was analyzed with a linear model using R [71], using time as a factor and male sex ratio as a variable. The total number of individuals present in one-yr old colonies was compared between the four mating combinations using ANOVA (HSD post-hoc). Proportions of castes and developmental stages comparison between colonies of the four mating combinations was done using a *χ* ^*2*^ test of independence.

## Supporting Information

S1 Fig Picture of tandem behavior between a female *C. formosanus* (left, large bright orange abdomen) and a male *C. gestroi* (right, small nutty brown abdomen) in the field. Picture taken on May 21st 2014 at 8:32pm during a simultaneous swarm, 1.5 m away from the light trap used in the experiment. The two dealates were looking for a suitable nesting site while walking on a piece of spruce that was placed on top of the plastic tarp. Under poor lighting condition and moving objects, it was difficult to obtain sharp macro photography, but the clear difference of morphology allowed for immediate species identification. (Picture: T.C.).
(TIF)

S2 Fig Genotyping for the microsatellite locus *Cg33* for 8 colonies (female, male, 9 workers and one soldier). Hybrid offspring inherited specific alleles from their respective *C. gestroi* and *C. formosanus* parents.
(TIF)

S3 Fig Genotyping for the microsatellite locus *Clac1* for 8 colonies (female, male, 9 workers and one soldier). Hybrid offspring inherited specific alleles from their respective *C. gestroi* and *C. formosanus* parents.
(TIF)

S4 Fig Assessment for the risk of introgressive hybridization. Our result supports that all pre-zygotic barriers are lifted, however the viability and fertility of F_1_ alates needs to be confirmed. Monitoring for introgression back to parental populations is needed.
(TIF)

S1 Table COII genotypes of individuals from endogamous and exogamous colonies (2 replicates per mating combination). Hybrid offspring inherited the maternal mitochondrial marker.
(DOCX)

S2 Table Microsatellites tested against *C. gestroi* and *C. formosanus* conspecific and heterospecific incipient colonies. (DOCX)
