# Supplementary figures and images for: Hybridization of Two Major Termite Invaders as a Consequence of Human Activity
Source: PLoS One. 2015 Mar 25;10(3):e0120745. doi: 10.1371/journal.pone.0120745 (PMC4373762; doi:10.1371/journal.pone.0120745)

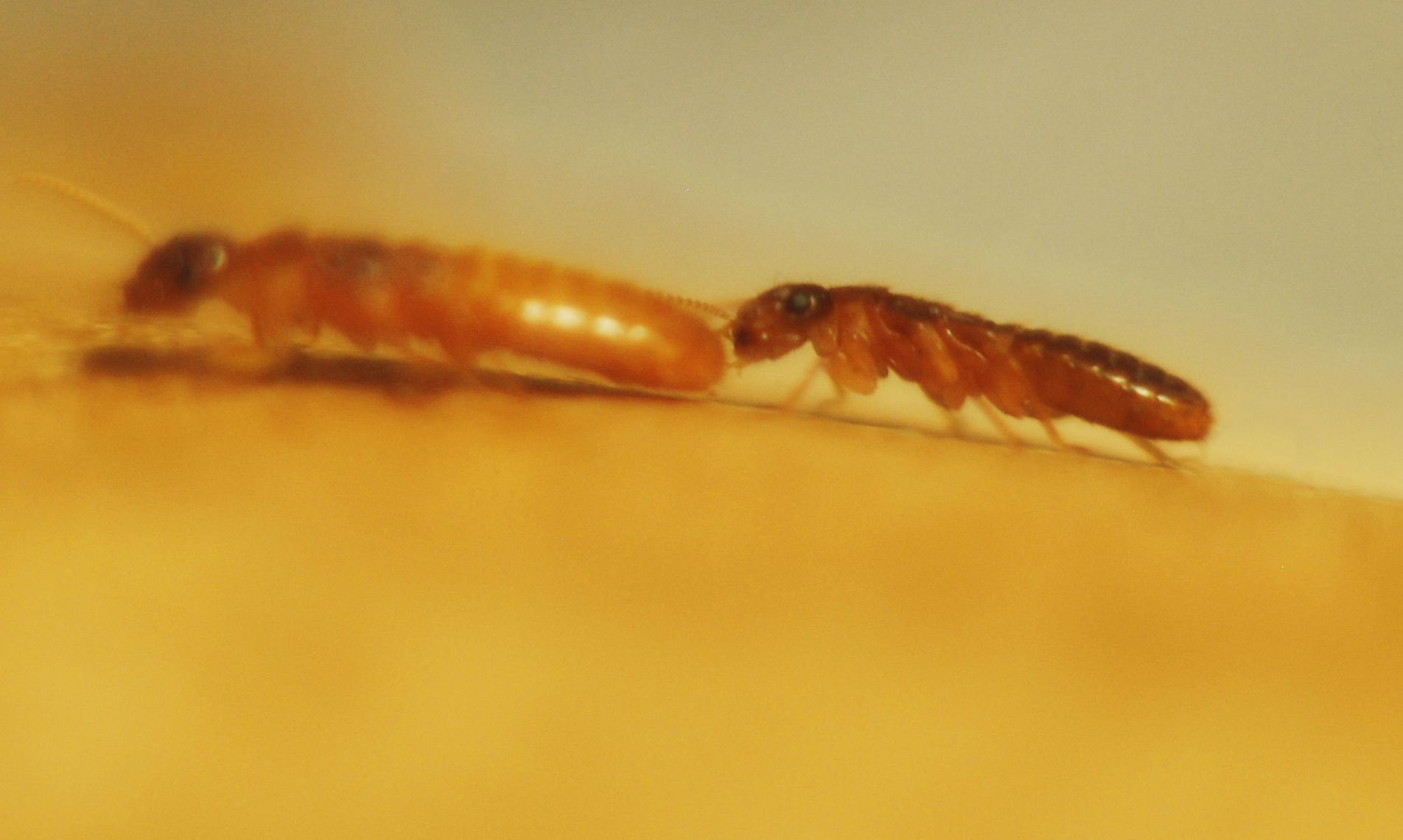

Supplement: S1 Fig — Picture taken on May 21st 2014 at 8:32pm during a simultaneous swarm, 1.5 m away from the light trap used in the experiment. The two dealates were looking for a suitable nesting site while walking on a piece of spruce that was placed on top of the plastic tarp. Under poor lighting condition and moving objects, it was difficult to obtain sharp macro photography, but the clear difference of morphology allowed for immediate species identification. (Picture: T.C.). (TIF) [file pone.0120745.s001.tif]

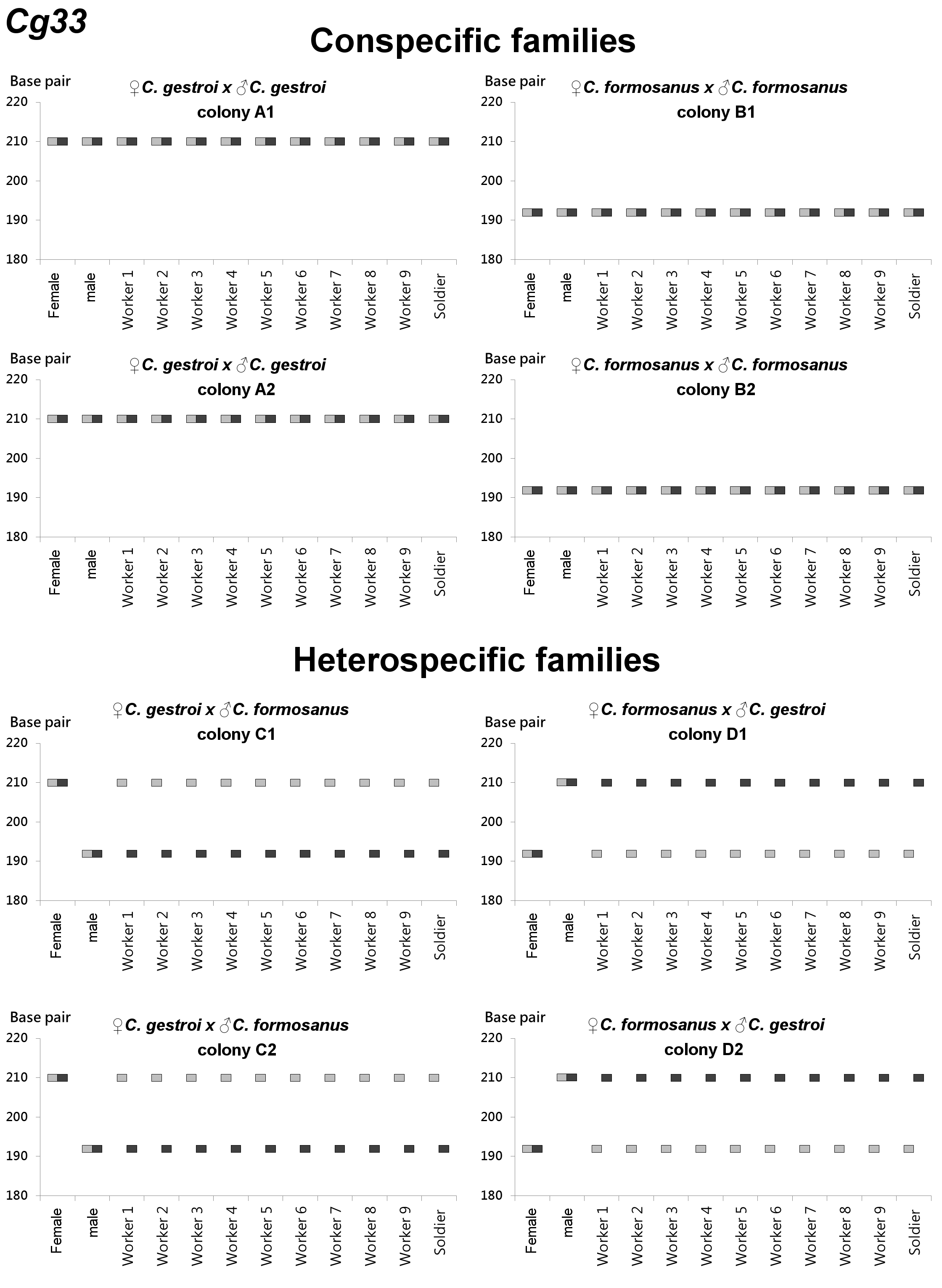

Supplement: S2 Fig — Hybrid offspring inherited specific alleles from their respective C. gestroi and C. formosanus parents. (TIF) [file pone.0120745.s002.tif]

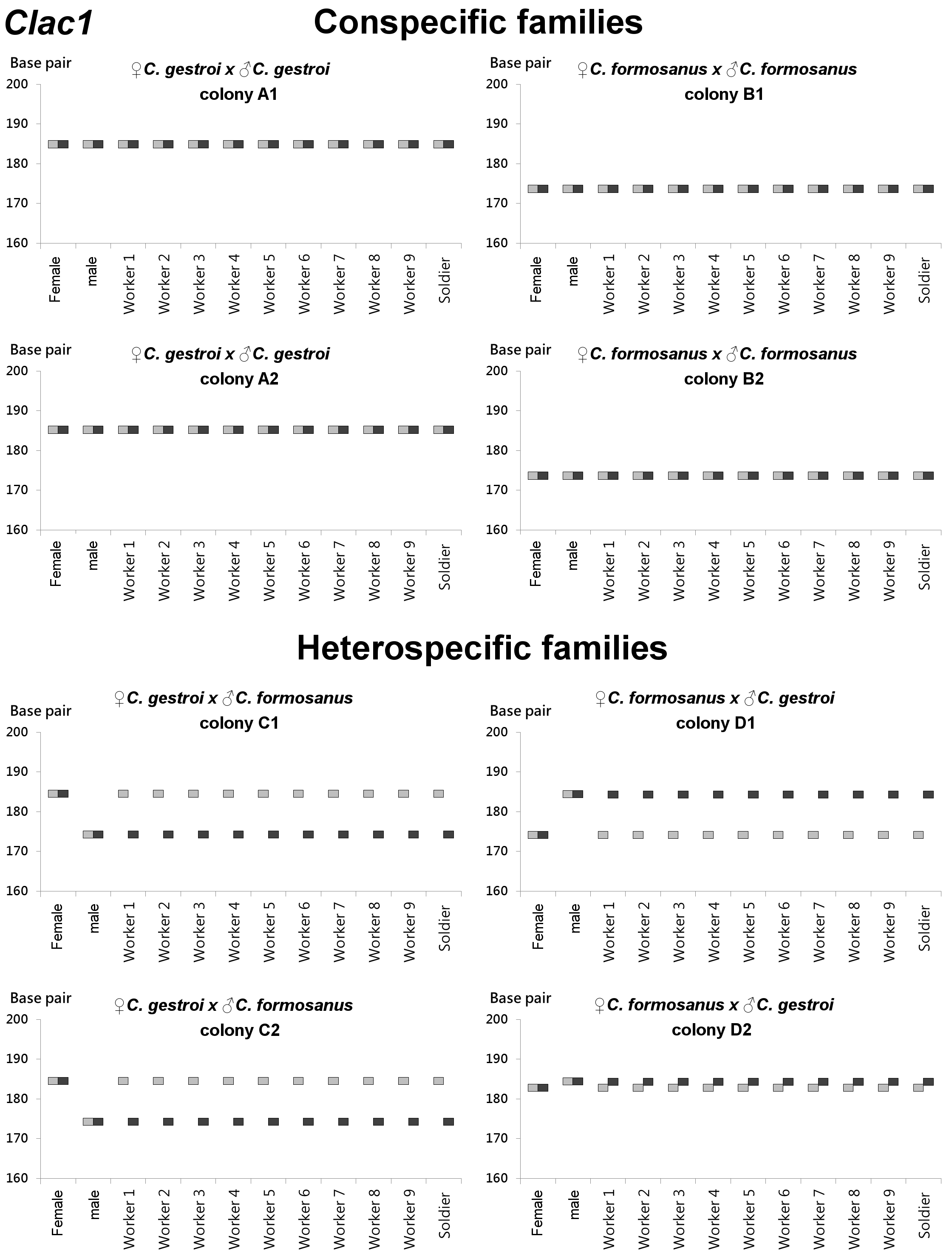

Supplement: S3 Fig — Hybrid offspring inherited specific alleles from their respective C. gestroi and C. formosanus parents. (TIF) [file pone.0120745.s003.tif]

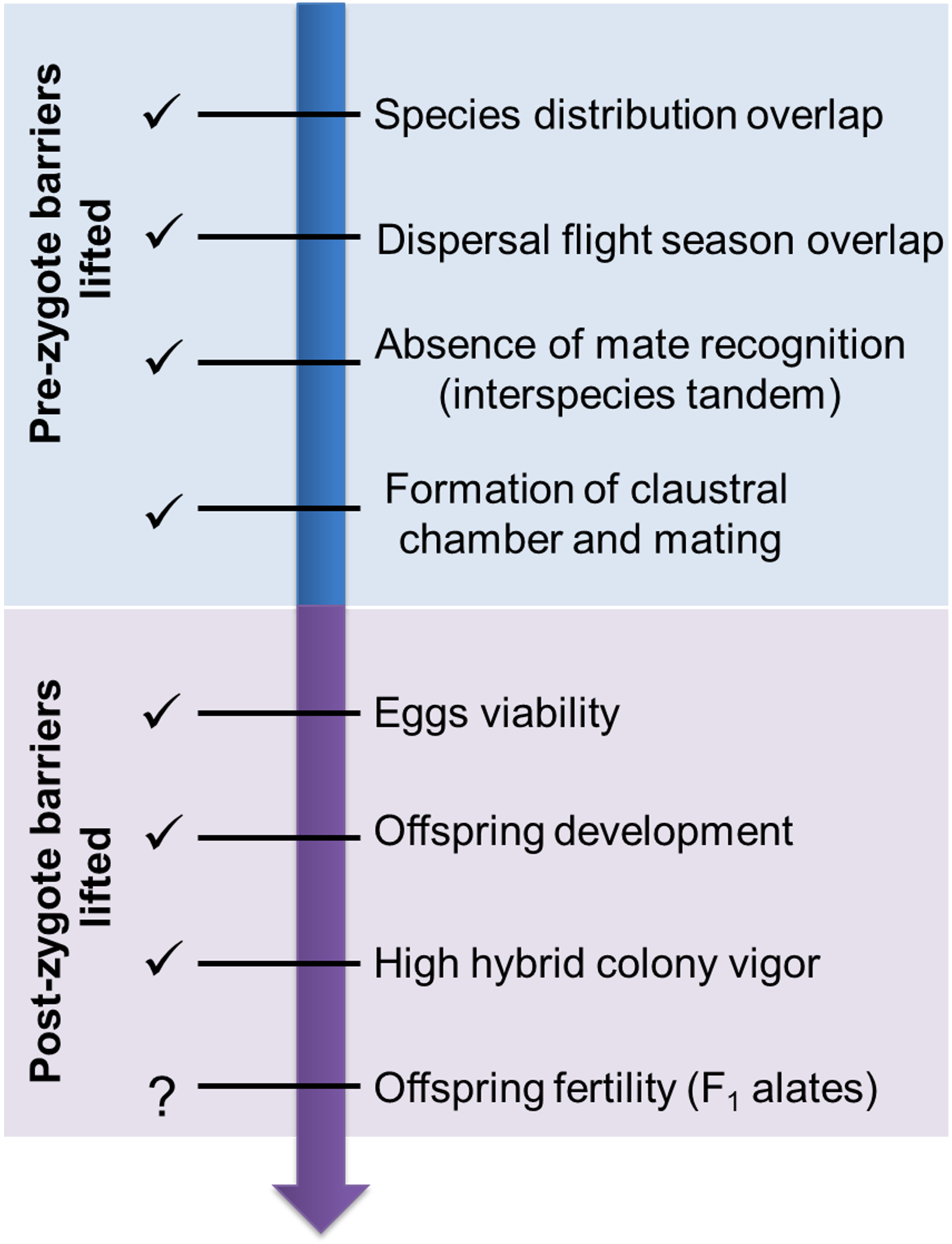

Supplement: S4 Fig — Our result supports that all pre-zygotic barriers are lifted, however the viability and fertility of F1 alates needs to be confirmed. Monitoring for introgression back to parental populations is needed. (TIF) [file pone.0120745.s004.tif]
