# Supplementary material for: Hybridization of Two Major Termite Invaders as a Consequence of Human Activity
Source: PLoS One. 2015 Mar 25;10(3):e0120745. doi: 10.1371/journal.pone.0120745 (PMC4373762; doi:10.1371/journal.pone.0120745)
Supplement: S1 Table — Hybrid offspring inherited the maternal mitochondrial marker. (DOCX) [file pone.0120745.s005.docx]

**Table S1.** COII genotypes of individuals from endogamous and exogamous colonies (2 replicates per mating combination). Hybrid offspring inherited the maternal mitochondrial marker.

| Conspecific families | | | | |  | Heterospecific families | | | | |  |
| --- | --- | --- | --- | --- | --- | --- | --- | --- | --- | --- | --- |
|  | |  | *C. gestroi* | *C. formosanus* |  |  | |  | *C. gestroi* | *C. formosanus* | |
| Colony | |  | COII | COII |  | Colony | |  | COII | COII | |
| of origin | |  | EU805757* | EU805770* |  | of origin | |  | EU805757* | EU805770* | |
|  | |  |  |  |  |  | |  |  |  |  |
| ♀*C. gestroi* x ♂*C. gestroi* A1 | | | | |  | ♀*C. gestroi* x ♂*C. formosanus* C1 | | | | |  |
|  | Queen | | x |  |  |  | Queen | | x |  |  |
|  | King | | x |  |  |  | King | |  | x |  |
|  | Workers 1-9 | | x |  |  |  | Workers 1-9 | | x |  |  |
|  | Soldier | | x |  |  |  | Soldier | | x |  |  |
|  |  | |  |  |  |  |  | |  |  |  |
| ♀*C. gestroi* x ♂*C. gestroi* A2 | | | | |  | ♀*C. gestroi* x ♂*C. formosanus* C2 | | | | |  |
|  | Queen | | x |  |  |  | Queen | | x |  |  |
|  | King | | x |  |  |  | King | |  | x |  |
|  | Workers 1-9 | | x |  |  |  | Workers 1-9 | | x |  |  |
|  | Soldier | | x |  |  |  | Soldier | | x |  |  |
|  |  | |  |  |  |  |  | |  |  |  |
| ♀*C. formosanus* x ♂*C. formosanus* B1 | | | | |  | ♀*C. formosanus* x ♂*C. gestroi* D1 | | | | |  |
|  | Queen | |  | x |  |  | Queen | |  | x |  |
|  | King | |  | x |  |  | King | | x |  |  |
|  | Workers 1-9 | |  | x |  |  | Workers 1-9 | |  | x |  |
|  | Soldier | |  | x |  |  | Soldier | |  | x |  |
|  |  | |  |  |  |  |  | |  |  |  |
| ♀*C. formosanus* x ♂*C. formosanus* B2 | | | | |  | ♀*C. formosanus* x ♂*C. gestroi* D2 | | | | |  |
|  | Queen | |  | x |  |  | Queen | |  | x |  |
|  | King | |  | x |  |  | King | | x |  |  |
|  | Workers 1-9 | |  | x |  |  | Workers 1-9 | |  | x |  |
|  | Soldier | |  | x |  |  | Soldier | |  | x |  |

*COII sequences are identical from the Florida *C. gestroi* and *C. formosanus* described by Li et al. [32]
