# Supplementary material for: Hybridization of Two Major Termite Invaders as a Consequence of Human Activity
Source: PLoS One. 2015 Mar 25;10(3):e0120745. doi: 10.1371/journal.pone.0120745 (PMC4373762; doi:10.1371/journal.pone.0120745)
Supplement: S2 Table — (DOCX) [file pone.0120745.s006.docx]

**Table S2**. Microsatellites tested against *C. gestroi* and *C. formosanus* conspecific and heterospecific incipient colonies.

| **Primer** | **Repeat Unit and Size** | **Reference** |
| --- | --- | --- |
| Cg6F/R | (GT)_12_; 160-188bp | [68] |
| Cg19F/R | (GT)_22_; 198-210bp |  |
| Cg31F/R | (GT)_5_; 150-170bp |  |
| Cg33F/R* | (CAA)_16_; 185-224bp |  |
| Cg38F/R | (GACA)_18_; 218-254bp |  |
| Cf10-5F/R | (GAT)_8_; 295bp | [69] |
| Cf4:1A2-4F/R | (AAG)_14_; 175bp |  |
| Cf1-1F/R | (TTA)_7_(GTA)_11_; 255bp |  |
| Cf4-4F/R | (ACT)_20_; 214bp |  |
| Cf4-9AF/R | (TCA)_11_; 283bp |  |
| Clac1F/R* | (AG)_9_AA(AG)_4_; 180-188bp | [70] |
| Clac8F/R | (GA)_10_; 249-258bp |  |

***** Loci that successfully yielded a single, but different sized allele for each species (specific homozygous alleles). These microsatellites were used for further analyses of all incipient colony mating combinations.
